# Continuing professional development ‐ Radiation Therapy

**DOI:** 10.1002/jmrs.655

**Published:** 2023-02-08

Maximise your CPD by reading the following selected article and answer the five questions. Please remember to self‐claim your CPD and retain your supporting evidence. Answers will be available via the QR code and online at www.asmirt.org/news-and-publications/jmrs, as well as published in JMRS – Volume 70, Issue 4 December 2023.

## Radiation Therapy – Original Article

### Clinical evaluation of deep learning and atlas‐based auto‐segmentation for critical organs at risk in radiation therapy

Gibbons E, Hoffmann M, Westhuyzen J, Hodgson A, Chick B, Last A. (2023) *J Med Radiat Sci*. https://doi.org/10.1002/jmrs.618

1. How are deep learning (DL) auto‐segmentation contours produced on an incoming data set?
  - DL models use deformable image registration to transform contours from a pre‐defined library of similar data sets
  - DL models predict contour location using optimised convolution ‘neural’ networks (CNNs) that have been trained to identify complex non‐linear spatial relationships within data sets
  - DL models generate contours by estimating spatial position on a data set using the Dice similarity coefficient (DSC) formula
  - DL models require users to roughly contour the incoming data set by hand before the algorithm can fully optimise each organ at risk (OAR)
2. Which of the following is correct in terms of the number of training data sets used for atlas and DL segmentation?
  - Atlas‐based auto‐segmentation typically uses a larger number of training data sets than DL models
  - If an atlas and DL auto‐segmentation model are trained using the same number of data sets, they are likely to output identical contours
  - The larger number of training data sets used in DL segmentation naturally incorporates a more diverse range of patient anatomy than atlas‐based methods
  - DL auto‐segmentation models typically reach a performance plateau when trained on 10–30 data sets
3. Which DL‐based OARs experienced statistically significant time improvements over atlas‐based OARs?
  - Spinal Cord, Rectum, Bladder, Left Femoral Head
  - Spinal Cord, Oral Cavity, Left Lung, Oesophagus, Heart
  - Left Parotid, Spinal Cord, Left Lung, Rectum, Bladder, Left Femoral Head
  - Left Parotid, Rectum, Bladder, Left Femoral Head
4. For auto‐segmented OAR contours, it is recommended that:
  - All DL generated OARs should be reviewed by an expert clinician prior to clinical use
  - All DL generated OARs are accurate enough to be used clinically without human review
  - Only a specific list of DL generated OARs requires expert review prior to clinical use
  - Only a specific list of atlas generated OARs require expert review prior to clinical use
5. Why can vendor trained ‘generic’ DL models be beneficial to a radiation therapy workflow?
  - They will always output contours that are quantitatively more accurate than a DL model trained by an individual department
  - They are pre‐optimised to output contours that will meet the specific contouring protocols for all departments worldwide
  - The output contours will always require less editing time than a DL model trained by an individual department
  - They can output quality contours without the time, data or resource requirements needed for departments to individually train their own DL models

## Answers


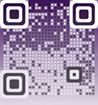


Scan this QR code to find the answers, or visit www.asmirt.org/news-and-publications/jmrs
